# Intimointimal intussusception in acute aortic dissection: a rare phenomenon

**DOI:** 10.1007/s12471-025-01933-8

**Published:** 2025-02-04

**Authors:** Gijs J. van Steenbergen, Rutger Brouwers, Erwin Tan

**Affiliations:** 1 Department of Cardiology, Catharina Hospital, Eindhoven, The Netherlands; 2 Department of Cardiothoracic Surgery, Catharina Hospital, Eindhoven, The Netherlands

A woman in her 60s presented with decreased consciousness and respiratory distress after being found collapsed at home. She exhibited a systolic blood pressure difference of > 50 mm Hg between arms. Echocardiography performed in the Emergency Department revealed pericardial effusion, a transvalvular mobile structure through the aortic valve, and aortic regurgitation (Fig. 1a, b). Transoesofagal echocardiography confirmed the suspicion of aortic dissection, disclosing the unique feature on intimointimal intussusception—where the dissected intima prolapses through the aortic valve into the left ventricular outflow tract (LVOT) (Fig. 1c; see video 1 (Electronic Supplementary Material); [1]). Due to hemodynamic instability, the patient was not suitable for pre-operative computed tomography scan and was directly transferred for salvage sternotomy to relieve the cardiac tamponade. Intraoperative findings included a circumferential transverse intimal tear halfway along the ascending aorta, extending to the aortic valve which is characteristic for intimointimal intussusception. Following emergent ascending aorta replacement and aortic valve resuspension, the patient suffered extensive cerebral ischemia, leading to her passing.

**Fig. 1:**
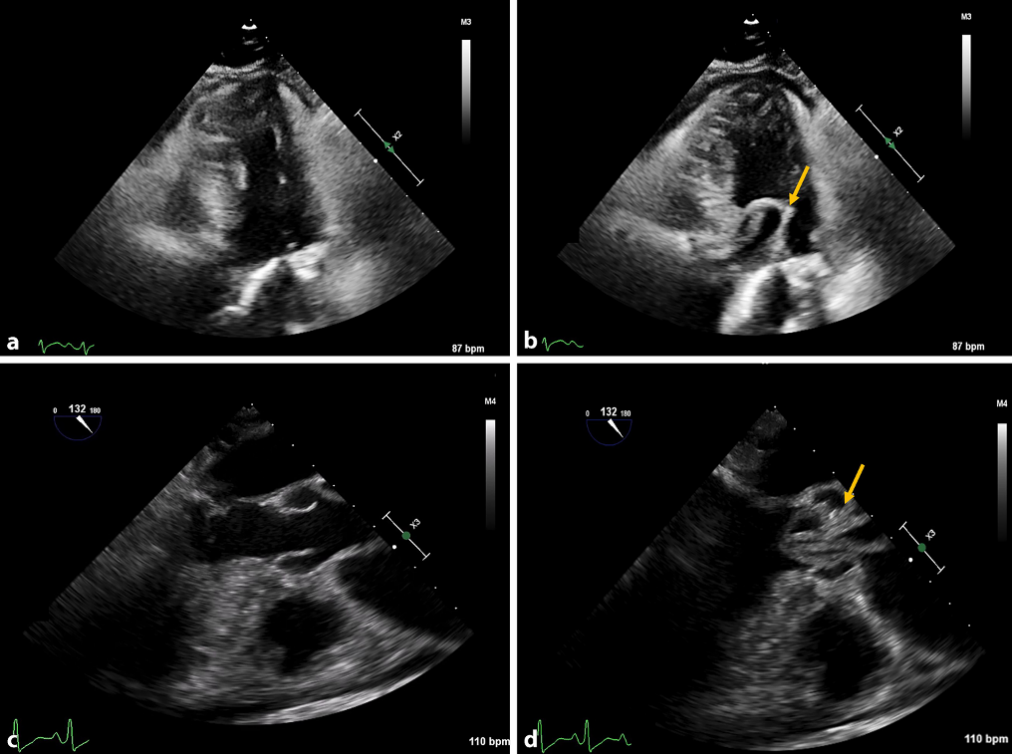
Echocardiography images of intimointimal intussusception. **a** Transthoracic echocardiography five-chamber view, systolic phase. **b** Transthoracic echocardiography five-chamber view, diastolic phase. **c** Transesophageal echocardiography 132°, systolic phase. **d** Transesophageal echocardiography 132°, diastolic phase. Yellow arrow indicates the intimointimal intussusception

This case underscores the importance of comprehensive echocardiographic evaluation in diagnosing aortic dissection, highlighting intimointimal intussusception as a critical, albeit rare, diagnostic clue.

## Supplementary Information

**video 1:** Transesophageal echocardiography at 132°
